# The Imbalance between n-6/n-3 Polyunsaturated Fatty Acids and Inflammatory Bowel Disease: A Comprehensive Review and Future Therapeutic Perspectives

**DOI:** 10.3390/ijms18122619

**Published:** 2017-12-05

**Authors:** Eleonora Scaioli, Elisa Liverani, Andrea Belluzzi

**Affiliations:** Department of Medical and Surgical Sciences, St. Orsola-Malpighi Hospital, University of Bologna, 40138 Bologna, Italy; elescaio@gmail.com (E.S.); elisa.liverani5@studio.unibo.it (E.L.)

**Keywords:** inflammatory bowel disease, ulcerative colitis, Crohn’s disease, n-3 polyunsaturated fatty acids, omega-3 fatty acids

## Abstract

Eating habits have changed dramatically over the years, leading to an imbalance in the ratio of n-6/n-3 polyunsaturated fatty acids (PUFAs) in favour of n-6 PUFAs, particularly in the Western diet. Meanwhile, the incidence of inflammatory bowel disease (IBD) is increasing worldwide. Recent epidemiological data indicate the potential beneficial effect of n-3 PUFAs in ulcerative colitis (UC) prevention, whereas consumption of a higher ratio of n-6 PUFAs versus n-3 PUFAs has been associated with an increased UC incidence. The long-chain dietary n-3 PUFAs are the major components of n-3 fish oil and have been shown to have anti-inflammatory properties in several chronic inflammatory disorders, being involved in the regulation of immunological and inflammatory responses. Despite experimental evidence implying biological plausibility, clinical data are still controversial, especially in Crohn’s disease. Clinical trials of fish-oil derivatives in IBD have produced mixed results, showing beneficial effects, but failing to demonstrate a clear protective effect in preventing clinical relapse. Such data are insufficient to make a recommendation for the use of n-3 PUFAs in clinical practice. Here, we present the findings of a comprehensive literature search on the role of n-3 PUFAs in IBD development and treatment, and highlight new therapeutic perspectives.

## 1. Introduction

Eating habits have dramatically changed over the years, leading to an imbalance in the ratio of n-6/n-3 polyunsaturated fatty acids (PUFAs) in favour of n-6 PUFAs, particularly in the Western diet [1]. These changing alimentary habits have coincided with a worldwide increase in the incidence of inflammatory bowel disease (IBD) in recent decades [2]. Consumption of a higher ratio of pro-inflammatory n-6 PUFAs, such as linoleic acid (LA) and arachidonic acid (AA), to n-3 PUFAs has been associated with an increased incidence of ulcerative colitis (UC) [3,4].

Recent epidemiological data indicate the potential beneficial effect of n-3 PUFAs in IBD, particularly in UC where a high intake of n-3 PUFAs may lower the incidence of the disease [5].

The long chain dietary n-3 PUFAs, in particular eicosapentaenoic acid (EPA) and docosahexaenoic acid (DHA), are the major components of n-3 fish oil and have been shown to have anti-inflammatory properties in several chronic inflammatory disorders, such as asthma and rheumatoid arthritis [6]. EPA and DHA are definitively involved in the regulation of immunological and inflammatory responses [7]; they inhibit genes that start the inflammatory process [8] and alter the composition of cell membranes by displacing n-6 PUFAs, influencing lipid raft formation in cell signalling [9]. Some of these anti-inflammatory effects of n-3 PUFAs may be mediated by competition with n-6 PUFAs, because n-3 PUFAs act as a competitive substrate for the metabolism of n-6 PUFAs [10]. More recently, new metabolic pathways for n-3 PUFAs have been reported, leading to production of inflammatory resolving mediators called resolvins, defensins, and maresins [11,12]. Despite experimental evidence implying biological plausibility, clinical data on the benefits of n-3 PUFAs in IBD are still controversial and conflicting, especially in Crohn’s disease (CD) [13,14]. Clinical trials of fish-oil derivatives in IBD have produced mixed results, showing beneficial effects, such as a reduction of inflammation and decreased need for steroid therapy, but also failing to demonstrate a clear protective effect in preventing clinical relapse [15,16,17,18,19,20,21,22,23,24,25]. Such data are insufficient to make a recommendation for the use of n-3 PUFAs in clinical practice [13,14,26,27,28]. We have carried out a comprehensive literature search regarding the anti-inflammatory mechanisms of action of n-3 PUFAs and, specifically, their utilisation for dampening down chronic inflammatory activity in IBD, and we present a critical analysis of clinical trials with n-3 PUFAs in this field, highlighting new therapeutic perspectives.

### 1.1. IBD Epidemiology and Dietary Intake of n-6/n-3 PUFAs

The incidences of UC and CD are increasing worldwide, reaching 20 new cases per 100,000 person-years in Western countries [2], as well as in countries where IBD was previously thought to be uncommon [29]. Furthermore, data from epidemiological studies of migrants to higher-IBD-prevalence countries show an increasing incidence of IBD [30,31]. These findings support the hypothesis of an environmental trigger for IBD development in genetically susceptible individuals.

Among many possible environmental factors, diet is one of the most important. Eating habits have changed dramatically over the centuries, moving from a Paleolithic diet (prior to the agricultural revolution, and comprising primarily meat, fish, vegetables, and fruit) to a Western diet (during the modern, postindustrial-revolution era, with increased consumption of grains and refined sugars) [32].

Based on the premise that human genetics have scarcely changed over the last 3000 years, modern humans should genetically be better adapted to the diet of their Paleolithic ancestors [32]. However, dietary changes stemming from the agricultural revolution have markedly changed patterns of food consumption, leading to an “evolutionary discordance” that may have contributed to the dramatic increase in chronic inflammatory diseases seen over the last century [33,34,35].

Supporting this concept, a recent pooled cross-sectional study of 646 subjects undergoing elective outpatient colonoscopy found significantly lower levels of systemic inflammation and oxidative stress in those patients who followed a more Paleolithic- and Mediterranean-like diet, compared with other types of diets [36].

One of the most contrasting elements between the Paleolithic diet and the Western diet is the differing ratios of n-6 and n-3 PUFAs, with the former diet being in perfect balance (1:1), while the latter is deeply unbalanced in favour of n-6 PUFAs (20:1) [37].

Blasbalg et al. [1] perfectly illustrate the profound change in PUFAs ratio during the last century in favour of n-6 PUFAs; indeed, the estimated per capita consumption of soybean oil increased >1000 fold from 1909 to 1999. The percentage contribution of energy from the n-6 PUFA, LA, increased from 2.79% to 7.21% (*p* < 0.000001), whereas that of the n-3 PUFA, α linolenic acid (ALA), increased from 0.39% to 0.72% using the same model, and the ratio of LA to ALA increased from 6.4 in 1909 to 10.0 in 1999. The increased consumption of LA from soybean oil has likely decreased tissue concentrations of EPA and DHA during the 20th century, and the omega-3 index [38] (a direct measure of erythrocyte EPA + DHA as a percentage of total fatty acids) declined from 8.28% in 1909 to 3.84% in 1999.

Since the 1990s, researchers have sought to define a correlation between the intake of n-3 and n-6 PUFAs and the incidence of IBD. Initial studies were retrospective and/or case-controlled in design, with poor reliability [39,40,41]; however, four recent large, prospective, epidemiological studies have contributed to better defining the role of PUFAs intake in the incidence of IBD.

Tjonneland et al. [4] analysed data from food frequency questionnaires completed by more than 200,000 participants in a prospective, multicenter cohort study, the European Prospective Investigation into Cancer and Nutrition (EPIC), who were followed up for diagnosis of UC. A total of 126 participants developed UC (47% women) after a median follow up of 4 years. Each case was matched with four controls and the risk of disease calculated by quartile. The highest quartile of intake of the n-6 PUFA, LA, was associated with an increased risk of UC (odds ratio (OR) 2.49, 95% confidence interval (CI): 1.23–5.07; *p* = 0.01) in both genders. In contrast, increased dietary intake of the n-3 PUFA, DHA, was associated with a reduced risk of UC; for the highest quartile, the OR was 0.23 (95% CI: 0.06–0.97). In this study, the main finding was that the highest intake of the n-6 PUFA, LA, was associated with more than a doubling of the risk of developing UC. Findings on n-3 PUFAs were less compelling, which may be due to a reduced sample size because data on certain n-3 PUFAs were not available from one centre.

John et al. [42] investigated more than 25,600 participants living in Norfolk, UK, who completed seven-day food diaries, among whom 22 incident cases of UC were identified after a median follow-up time of 4.2 years. A statistically significant, dose-dependent, negative association was found between increased intake of DHA and the risk of developing UC. The highest tertile of DHA intake showed a reduction in risk of 83% (OR 0.17, 95% CI: 0.04–0.78; *p* = 0.02). Moreover, there were negative associations for UC with total n-3 PUFAs (OR 0.56, 95% CI: 0.28–1.13; *p* = 0.10) and EPA (OR 0.53, 95% CI: 0.27–1.03; *p* = 0.06), which were of borderline statistical significance.

Ananthakrishnan et al. [5] conducted a prospective study of women enrolled in the Nurses’ Health Study cohorts. Diet was prospectively ascertained every 4 years using a validated semi-quantitative food frequency questionnaire. Among 170,800 women, the authors confirmed 269 incident cases of CD and 338 incident cases of UC over 26 years, and 3,317,338 person-years of follow up. Intake of n-3 PUFAs (docosapentaenoic acid (DPA), EPA, and DHA) was inversely associated with risk of UC; those with the highest intake had reduced incidence of UC (hazard ratio (HR) 0.72, 95% CI: 0.51–1.02; *p*(trend) = 0.13). Moreover, compared to the lowest quintile of the ratio between n-3 and n-6 PUFAs, women in the highest quintile had an HR of 0.69 (95% CI: 0.49–0.98; *p*(trend) = 0.03) for UC. In contrast, intake of each individual fatty acid (either n-3 or n-6 PUFAs) was not associated with risk of CD.

Chan et al. [43] investigated 229,700 participants from the EPIC cohort, using a validated food frequency questionnaire that assessed dietary intake of DHA and other fatty acids, to identify those who developed incident CD. The cohort was recruited between 1991–1998 and monitored until June 2004 in a nested case-control analysis; each case was matched with four controls. Seventy-three participants developed CD. The highest quintile of DHA intake was inversely associated with development of CD (OR 0.07, 95% CI: 0.02–0.81). No other associations were found with respect to other dietary fatty acids.

Hou et al. [44] carried out a systematic review of the literature on dietary intake and the risk of developing IBD. Among 19 identified studies, the authors reported an increased risk of developing UC with high intake of total fat, n-6 PUFAs, and meat, as well as an increased risk of CD with a high intake of n-6 PUFAs, saturated fats, and meat. No evidence of a protective effect of dietary n-3 PUFAs pre-illness was found.

### 1.2. n-6/n-3 PUFAs and Inflammatory Processes in IBD

#### 1.2.1. Eicosanoid Pathways and Inflammation

n-3 and n-6 PUFAs are defined by the position of the first double bond in the carbon chain. The human body can produce all but 2 PUFAs: the essential fatty acids, LA, the precursor to the n-6 series, and ALA, the precursor to the n-3 series. Eicosanoids are key lipid mediators generated from the n-6 and n-3 PUFAs that have important roles in immune regulation and inflammation. They are synthesised from 20-carbon PUFAs using AA as the major substrate. Free AA is metabolised by three main classes of enzymes: (1) cyclooxygenases (COX), which produce prostaglandins (PG) and thromboxanes (TX); (2) lipoxygenases (LOX), which generate leukotrienes (LT), hydroxyeicosatetraenoic acid (HETEs), and lipoxins (LX); and (3) p450 epoxygenases, which synthesise epoxyeicosatrienoic acids (EETs). These eicosanoids are deeply involved in the inflammatory processes of IBD; they have a potent chemotactic action and an ability to recruit neutrophils, increase vascular permeability, promote platelet aggregation, cause oedema, and induce the release of pro-inflammatory substances, such as cytokines (soluble proteins influencing the immune system), which lead to the production of chronic inflammatory mediators, such as interleukin (IL) 1β, 6, and 8, and tumor necrosis factor α (TNFα) [11,45].

Findings suggest that the colonic mucosa in active UC are associated with a marked increase in the availability of n-6 PUFAs, specifically AA (*p* < 0.001); conversely, n-3 PUFAs, specifically EPA, are less represented (*p* < 0.01) and the ratio of AA/EPA is higher (*p* < 0.001) with respect to controls [46].

n-3 PUFAs also serve as precursors of a class of eicosanoids with very little or no inflammatory properties, such as LTB5 [47].

It has also been widely demonstrated that n-3 PUFAs such as EPA and DHA are able to inhibit inflammatory cytokine production [48]. The incorporation of EPA and DHA into human inflammatory cells occurs in a dose-responsive fashion and partly displaces AA [49].

Dietary supplementation with n-3 PUFAs results in decreased production of PGE2, TXB2, and HETEs [11].

**Figure 1:**
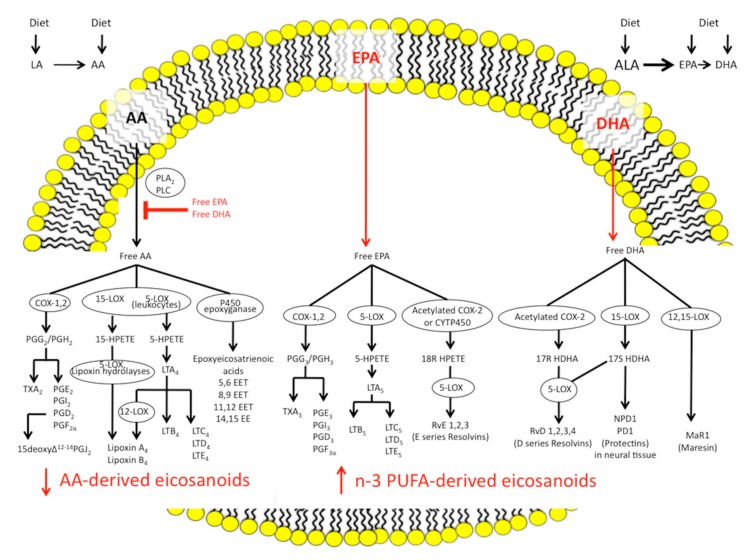
Outline of the pathways of synthesis for the n-3 and n-6 polyunsaturated fatty acids eicosanoids and the specialised pro-resolving mediators. AA, arachidonic acid; ALA, α-linolenic acid; COX, cyclooxygenase; CYTP450, cytochrome P450 enzymes; DHA, docosahexaenoic acid; EET, epoxyeicosatrienoic acid; EPA, eicosapentaenoic acid; HDHA, dihydroxy-docosahexaenoic acid; HPETE, hydroperoxyeicosatetraenoic acid; LA, linoleic acid; LT, leukotriene; LOX, lipoxygenase; MaR, maresin; NPD1, neuroprotectin D1; PD1, protectin D1; PG, prostaglandin; PLA2, phospholipase A2; PLC, phospholipase C; PUFA, polyunsaturated fatty acid; RvD, D series resolvins; RvE, E series resolvins; TX, thromboxane.

Moreover, new n-3 PUFAs-derived anti-inflammatory molecules, collectively termed specialised pro-resolving mediators (SPMs) and including resolvins, protectins, and maresins, have been identified [12]. SPMs counteract pro-inflammatory chemical mediators, reducing the magnitude and duration of inflammation, increasing production of anti-inflammatory cytokines (i.e., IL10), and stimulating wound healing, tissue regeneration, and re-epithelialisation. Figure 1 summarises the pathways of synthesis for the n-3 and n-6 PUFAs eicosanoids and the SPMs.

#### 1.2.2. n-3 PUFAs and the Endocannabinoid Epoxides Pathway

A very recent study reported on endogenous production of a previously unknown class of n-3 PUFAs-derived lipid metabolites, originating from cross-talk between endocannabinoid and cytochrome P450 (CYTP450) oxygenase metabolic pathways [50]. The n-3 endocannabinoid epoxides have been shown to dose-dependently decrease production of the pro-inflammatory cytokine, IL6, while increasing production of the anti-inflammatory cytokine, IL10. Moreover, n-3 endocannabinoid epoxides may exert anti-angiogenic effects in human microvascular endothelial cells. Taken together, these newly discovered n-3 endocannabinoid epoxides are expected to play a critical role during inflammation in vivo.

#### 1.2.3. n-3 PUFAs and Inflammatory Gene Expression

The two main transcription factors involved in IBD inflammatory processes are nuclear factor κB (NFκB), which is proinflammatory, and peroxisome proliferator activated receptor gamma (PPAR-γ), which is antinflammatory; both are expressed in the colon. PPAR-γ regulates inflammation by inhibiting NFκB [51,52]. Reduced expression of PPAR-γ has been reported in patients with UC and CD [53]. Many of the effects of n-3 PUFAs on the production of inflammatory mediators appear to be related to altered expression of genes encoding these mediators. n-3 PUFAs may modify the activity of the transcription factors NFκB and PPAR-γ, in particular, by promoting PPAR-γ gene transcription and inhibiting NFκB activation [54,55].

Knock et al. [56] compared the effect of EPA supplementation with that of oleic acid (OA) as a control in IL10-encoding gene-deficient mice. Mice fed an OA-supplemented diet had decreased expression of genes encoding antioxidant enzymes, as well as enzymes involved in detoxification, when compared with wild-type mice on the same diet. Conversely, EPA supplementation up-regulated the expression of these same enzymes, showing a potent anti-inflammatory effect on colon tissue.

Costea et al. [57] investigated whether variation in genes that control n-3 PUFAs metabolism (CYP4F3, FADS1, FADS2), together with alterations in the dietary ratio of n-6/n-3 PUFAs, confer susceptibility to CD. One-hundred and eighty-two children newly diagnosed with CD and 250 controls were studied, investigating 15 single nucleotide polymorphisms (SNPs) across these 3 genes. Logistic regression analysis showed that a higher ratio of n-6/n-3 PUFAs was associated with increased risk of CD (OR 1.63, 95% CI: 1.01–2.62; *p* = 0.044). The investigators observed a significant interaction (*p* < 0.05) involving SNPs, suggesting that the association between dietary n-6/n-3 PUFAs ratio and CD was influenced by the presence of specific variants of CYP4F3 and FADS2. The CYP4F3 gene is expressed in neutrophils, monocytes, and intestine, and is an important modulator of the inflammatory process via its ability to inactivate LTB4 [58]. The ability of CYTP450 F3 to detoxify those fatty acids that are intimately associated with inflammation makes it a relevant candidate for the treatment of CD. The FADS2 gene is a key PUFAs metabolic gene and desaturase that catalyses the first step in the conversion of essential PUFAs such as n-6 LA and n-3 ALA into longer chain n-6 and n-3 PUFAs. It has been observed that variations in this gene are associated with endogenous serum/plasma levels of EPA, DPA, DHA, and different PUFAs ratios [59]. The elevated risk for CD associated with increased dietary ratio of n-6/n-3 PUFAs in those individuals with particular FADS2 SNPs indeed suggests that lower endogenous production of n-3 PUFAs, combined with a higher n-6/n-3 dietary ratio, may increase the risk of CD in children [57].

Zhang et al. [60] reviewed the mechanism involved in the endogenous synthesis of long-chain PUFAs and showed that this is strongly dependent on the action of the fatty acid desaturase (FADS) and elongase (ELOVL) enzymes. Polymorphisms in the FADS and ELOVL genes can limit accumulation of long-chain PUFAs. The genes mediating the endogenous synthesis of PUFAs contribute to wide variability in the efficiency of this process, likely influenced by variation in FADS and ELOVL genes, as well as the metabolic state. In the current era of mass individual migration and easy availability of imported international foodstuffs, individuals with genotypes adapted to a particular diet that contains high or low amounts of long-chain PUFAs may find themselves exposed to other diets with a different PUFAs content than they are used to. In these cases, optimum nutrition depends on the detailed genetic control and efficiency of the process of long-chain PUFAs conversion.

Grimble et al. [61] investigated the relationship between TNFα gene polymorphisms and n-3 PUFAs. TNFα production varies widely among healthy individuals, and this study showed that the ability of n-3 PUFAs to suppress TNFα production from peripheral blood mononuclear cells in healthy men is deeply influenced by inherent TNFα production and TNFα gene polymorphisms. TNFα production decreased significantly after fish-oil supplementation in subjects who had a higher basal level of TNFα production, whereas the effects of dietary n-3 PUFAs intake were much less pronounced in subjects with lower basal TNFα production and paradoxically increased in those in the lowest tertile of basal TNFα production.

#### 1.2.4. n-3 PUFAs and Adaptive Immunity

n-3 PUFAs are able to modulate chemotaxis of immune cells, specifically a time-dependent decrease in chemotaxis of human neutrophils and monocytes towards various chemoattractants, including LTB4 [62].

Moreover, cell culture and animal feeding studies reported decreased expression of some adhesion molecules, such as vascular cell adhesion molecule 1 (VCAM-1), on the surfaces of monocytes, macrophages, lymphocytes, and endothelial cells following exposure to marine n-3 PUFAs [63]. This effect was independent of eicosanoid production and antioxidant status [64].

#### 1.2.5. n-3 PUFAs, Innate Immunity, the Inflammasome, and Microbiome

IBD patients exhibit aberrant innate immunity [65], as well as abnormal gut microbiome composition and activity [66].

The family of membrane-bound Toll-like receptors (TLRs) are considered to be an interface between the intestinal epithelial barrier, microbiota, and the immune system: TLR signalling pathways can be activated by microbial pathogens, while genetic defects in TLRs can cause a breakdown in tolerance to normally non-pathogenic intestinal microbiota in genetically predisposed individuals [67]. Both situations can be involved in IBD pathogenesis.

Nucleotide-binding oligomerisation domain 2 (NOD2), which is an intracellular sensor of bacterial peptidoglycan, was identified as a gene associated with susceptibility to CD [68].

It is postulated that the ability of CD-associated variants of NOD2 to recognise microbial components is likely to be impaired to varying degrees, resulting in the inappropriate activation of NFkB in monocytes [69]. Both TLR and NOD2 signalling are involved in increased intestinal permeability and alteration of tight junction proteins.

In vitro evidence suggests that certain types of fatty acids modulate NOD and TLR-mediated inflammation. Saturated fatty acid and n-6 PUFAs may exacerbate intestinal inflammation by upregulating TLR and NOD pathways, and increase intestinal permeability as a result of an alteration of tight junction protein [70]. n-3 PUFAs modulate membrane receptors and, in particular, strongly inhibit the expression of TLR4 [71] and dampen NOD2 signalling by blocking the release of NFκB from a protein kinase pathway [mitogen-activated protein kinase (MAPK)] [72].

The inflammasome is a cytosolic protein complex composed of NOD-like receptors (NLRs) that is a central regulator of innate immunity and inflammation [73]. In response to microbial and danger signals, it promotes caspase 1 activation, leading to the release of several pro-inflammatory cytokines (i.e., IL1β, IL18).

In IBD patients, the NLRP3 inflammasome has been found to be chronically activated [74]. A high-fat diet activates the NLRP3 inflammasome in macrophages [75]; n-3 PUFAs, mainly EPA, are able to abolish NLRP3 inflammasome activation, thus inhibiting subsequent release of cytokines [76].

It is already established that a Western diet may influence intestinal microflora, producing dysbiosis [77], and a metabolomics study reported that metabolites produced by the gut microbiome in CD patients strongly correlated with the content of dietetic AA and LA [78]. Conversely, in an animal model [79,80] and in a small human study [81], the administration of n-3 PUFAs altered gut microbiome and improved dysbiosis through enrichment of Lactobacillus species and a reduction in bacteria of the Bacteroidaceae family.

Very recent findings show a negative association between Akkermansia abundance and levels of n-6 PUFAs [82]. Watson et al. [83] investigated the effect of n-3 PUFAs supplementation for 8 weeks on the fecal microbiome in 22 middle-aged, healthy volunteers, reporting a reversible increase in several short-chain fatty acid-producing bacteria (Bifidobacterium, Lachnospira, Roseburia, and Lactobacillus) without a significant change in microbial diversity.

**Figure 2:**
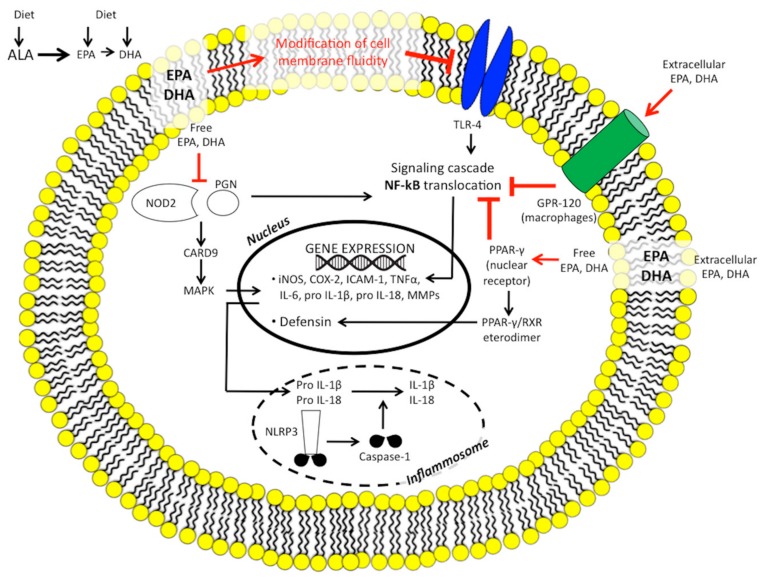
The n-3 polyunsaturated fatty acid targets of intestinal innate immunity. ALA, α-linolenic acid; CARD, caspase recognition domain; COX, cyclooxygenase; DHA, docosahexaenoic acid; EPA, eicosapentaenoic acid; GPR, G-protein coupled receptor; ICAM, intracellular adhesion molecule; IL, interleukin; iNOS, inducible nitric oxide synthase; MAPK, mitogen-activated protein kinase; MMPs, matrix metalloproteinase; NF-κB, nuclear factor kB; NLRP3, NOD-like receptor protein 3; NOD2, nucleotide-binding oligomerisation domain 2; PGN, bacterial peptidoglycan; PPAR-γ, peroxisome proliferator-activated receptor γ; RXR, retinoid X receptor; TLR, toll-like receptor; TNFα, tumour necrosis factor α.

These data support the concept that n-3 PUFAs may directly modulate innate immunity, whereas the evidence that supports their influence on microbiome composition is still preliminary, although encouraging. Figure 2 summarises the n-3 PUFAs targets of intestinal innate immunity.

#### 1.2.6. n-3 PUFAs and Nitric Oxide

Nitric oxide (NO) is a free radical synthesised from L-arginine by nitric oxide synthase (NOS) that is involved in many biological functions. Excessive amounts of NO lead to inflammation related-tissue damage, and NO has been proposed to play an important role in the inflammatory processes involved in the pathogenesis of UC [84]. n-3 PUFAs can act at the nuclear level to affect expression of genes involved in different metabolic pathways, and regulation of inducible NOS gene expression has been shown to be markedly decreased after administration of n-3 PUFAs, leading to a subsequent decrease in NO production [85].

### 1.3. n-6 and n-3 PUFAs in IBD Clinical Trials

To better understand the complexity of the disease and the need for well-designed trials in IBD, the first study we would mention is a double-blind, randomised, placebo-controlled trial in which both essential n-3 and n-6 PUFAs were administered together in a group of UC patients, with the aim of reducing the frequency of disease relapse. Middleton et al. [86] treated 63 UC patients with 6 capsules per day of n-6 gamma linolenic acid (GLA) together with EPA and DHA (500 mg each) versus 6 capsules daily of a sunflower oil placebo, itself a rich source of n-6 PUFAs. As it was possible to predict based on confounding premises, the study showed a similar relapse rate in the two groups after 12 months and no changes in sigmoidoscopic grade from baseline.

Despite the experimental evidence implying the biological plausibility of employing n-3 PUFAs for IBD, clinical data are still controversial and conflicting.

Investigations into IBD began at the end of the 1980s. The first evidence of clinical benefit of n-3 PUFAs came from McCall [18] who, in an open-label study, gave 3–4 g of EPA daily (16–24 capsules of fish oil as triacylglycerol) for 12 weeks to 6 patients with active UC. A significant improvement in symptoms and histological appearance was observed, along with a significant fall in neutrophil LTB4 production.

In the 1990s, Salomon et al. [20], in another open-label study, administered fish oil-derived n-3 PUFAs to 10 UC patients who were refractory to conventional treatment (steroids and salicylates), and obtained a significant improvement in all activity parameters in 7 of 10 patients. The first prospective, controlled, double-blind study was published by Lorenz et al. [23] who treated 39 patients with IBD (of whom 29 had CD) in different stages of clinical activity in a 7-month, placebo-controlled, crossover trial. Patients were randomised to either 3.2 g daily of n-3 PUFAs or olive oil as placebo. Conventional treatment was discontinued whenever possible or minimised to a constant low level for at least 3 weeks before the study until completion. There was a one-month wash out-period prior to switching treatments. At the end of the study, clinical activity expressed by Crohn’s Disease Activity Index (CDAI) [87] was unchanged in patients with CD after n-3 PUFAs supplementation. However, a limitation in the design of this cross-over study was the very short wash-out period between the two treatments. This would not allow a complete displacement of the extra n-3 PUFAs from cellular membranes and could have interfered with the final results of the study. It has previously been shown that inhibition of cytokine production by n-3 PUFAs persists for more than 10 weeks after suspension of treatment with n-3 PUFAs [48]. Hawthorne et al. [17] published the first large placebo-controlled study in 1992. In this study, 96 UC patients in different activity stages were enrolled and were given 4.5 g daily of EPA as triacylglycerol for 1 year, with the patients in the placebo group receiving olive oil. In patients with active disease at entry, it was possible to demonstrate a significant steroid-sparing effect, but fish oil failed to prevent clinical relapse in the group of patients who were enrolled in remission. Remarkably, LTB4 production in stimulated neutrophils was reduced by more than 50%. Stenson et al. [16] carried out a randomised, double-blind, placebo-controlled crossover study with 5.4 g of n-3 PUFA as triacylglycerol (18 capsules daily), or olive oil as placebo, in 24 patients with active UC.

The patients received treatment for 4 months followed by 1 month of washout. The study demonstrated that fish oil was able to induce a significant gain in body weight, significantly improve histology score, and reduce LTB4 production in rectal dialysates by 60%. No significant steroid-sparing effect was found, compared with placebo, and the improvement in endoscopy score did not reach significance (*p* = 0.06). Aslan et al. [15] carried out a similar placebo-controlled crossover trial in 17 patients with active UC, who received 4.2 g of n-3 PUFAs daily or corn oil as placebo for 3 months, followed by 2 months of wash-out. In 72% of patients, a steroid-sparing effect was seen, and in 56% the activity score of the disease improved significantly. Improvement of histology score did not reach statistical significance. Loeschke et al. [19] conducted a placebo-controlled trial on the prevention of UC relapse, in which 64 patients in remission were randomised to receive 5.1 g of n-3 PUFAs as ethyl esters in fish oil or maize oil as placebo; ongoing treatment with 5-aminosalicylic acid (5-ASA) was allowed for 3 months. Interestingly, after 3 months of study the fish-oil group had fewer relapses than the placebo group (*p* < 0.02), but this beneficial effect was lost by the end of the study (2 years). This leads to speculation that fish oil and 5-ASA may have synergistic effects, and also that patient compliance in the fish-oil group decreased during the study and could have affected the clinical outcome. Lorenz-Meyer et al. [25] published data from a large, placebo-controlled trial in 204 CD patients, who were included after an acute relapse of their disease, in which remission (CDAI < 150) was obtained under steroid therapy. Patients were randomised to either n-3 PUFAs (5.1 g daily of fish oil as ethyl esters; *n* = 70), a carbohydrate-reduced diet (72 g/daily; *n* = 69), or placebo (corn oil; *n* = 65) for 1 year. Low-dose prednisolone was given to all patients for the first 8 weeks of the trial and then discontinued. On an intent-to-treat analysis, none of the treatments were able to prevent clinical flare-up, but the diet poor in carbohydrates seemed to be effective at lowering the risk of relapse (*p* < 0.05), although it had the highest number of drop-outs (20 of 69 patients; 35%). Almallah et al. [21], in a pilot study, randomised 18 UC patients with distal procto-colitis to 5.6 g/day EPA + DHA and placebo (sunflower oil) in a double-blind manner for 6 months. At the end of the study, the n-3 PUFAs group showed improvement in clinical activity, and sigmoidoscopic and histological scores, compared with the placebo group.

Possible explanations for these disparate results could be related to questionable study design, particularly in the cross-over study, in which a short wash-out period between active treatment and placebo may likely not have allowed a complete displacement of the extra n-3 PUFAs from cell membranes and could have interfered with the final results. Other possible factors may have been the use of different formulations and doses of n-3 PUFAs, poor patient compliance, incorrect administration regimens, and choosing olive oil or other oils rich in n-6 PUFAs with a strong biological activity, such as corn, maize and sunflower oil, as a placebo [88,89].

Our group tested a new formulation of n-3 PUFAs, comprising a free fatty acid (FFA) mixture of 45% EPA and 20% DHA in enteric-coated capsules, in 78 CD patients in remission but with high risk of relapse according to criteria defined by Brignola et al. [90]. The patients were randomly assigned to receive daily capsules containing either 2.7 g of n-3 PUFAs or placebo (2.7 g capryl acid and capric acid). After 1 year of treatment, 59% of patients in the active group were still in remission, compared with only 26% in the placebo group (*p* = 0.006). Multivariate logistic regression analysis indicated that only n-3 PUFAs treatment reduced the likelihood of relapse (OR 4.2, 95% CI: 1.6–10.7) [91]. Feagan et al. [92] tested a similar preparation of n-3 PUFAs in a large cohort of quiescent CD patients (*n* > 700) from the EPIC-1 and EPIC-2 trials, in order to prevent clinical relapse at 12 months. The endpoint was not reached and active treatment did not reduce relapse rate in CD patients between groups; even when taking the whole population for virtually all levels of remission, there was a delay of 30–50 days for the active treatment versus placebo group. It is difficult to explain the limited or absent clinical benefit of n-3 PUFAs in these two CD trials, in view of the strong biological rationale and positive data from previous studies.

The results were negative with comparable low relapse rates between active (31.6%) and placebo (35.7%) groups in the EPIC-1 trial, and the active (47.8%) and placebo (48.8%) groups in the EPIC-2 trial, after 1 year of follow up. A conceivable explanation for this failure is the lower sensitivity of the main clinical inclusion criteria in identifying patients at high risk of relapse in EPIC-1; in fact, the main inclusion criterion was “previous time in remission”. The introduction of a reliable inflammatory marker of CD, such as C reactive protein (CRP) at entry, could have better stratified patients with high risk of relapse. A significant group of patients may have been in stable remission and at low risk of relapse, possibly accounting for the low relapse rate in the placebo group after 1 year (35.7% versus 65% in the Belluzzi study [91]).

EPIC-2 had a different design [90]. Relapse rate during follow-up after steroid treatment in the placebo group was 48.8% versus 47.8% in the active-treatment group, which is very low when compared to similar trials (up to 90%) [93]. Fewer than 2% of patients had previous treatment with biologicals and only 7% were treated with immuno-modifying agents, suggesting they had very mild disease. It is unclear how many patients had genuine relapses requiring steroid treatment (CDAI at entry not shown). CRP levels after steroid tapering were not reported, so, again, a “real risk” of subsequent relapse is unknown.

Romano et al. [94] conducted a study of 38 children with quiescent CD, in which the addition of 1.8 g daily of enteric-coated n-3 PUFAs to 5-ASA treatment for 12 months appeared to be of benefit versus identical olive oil-containing placebo, in terms of number of clinical relapses (*p* < 0.001).

In a very interesting study, Uchiyama et al. [95] investigated the ability of a diet rich in n-3 PUFAs to rebalance the n-6/n-3 PUFAs ratio in patients with IBD. Because the 1:1 ratio target was too difficult to reach, the authors chose to aim for a ratio of 2:1 n-6/n-3 PUFAs. This was achieved using combined dietary changes (about 1700 mg/day from EPA and DHA) plus supplementation of 7 mL/day of perilla oil (about 3400 mg/day of ALA), and fatty acid composition of the erythrocyte membranes of 20 initial-onset IBD patients was measured. The authors found a significant increase in mean n-3/n-6 PUFAs ratio after 12 months of intervention (0.41 ± 0.16 versus 0.70 ± 0.20; *p* < 0.001). Furthermore, an additional 230 IBD patients (168 UC and 62 CD) after clinical remission underwent this n-3 PUFAs diet therapy plus perilla oil regimen. During the 18 months follow-up period, the n3-/n-6 PUFAs ratio in patients in the remission group (*n* = 145) was significantly higher than that of patients in the relapse group (*n* = 85) (0.65 ± 0.28 versus 0.53 ± 0.18; *p* < 0.001), confirming that alteration of cell membrane fatty acid composition after supplementation of n-3 PUFAs may have an influence on clinical activity of the disease.

Keeping in mind the importance of having a reliable marker of subclinical inflammation (an objective predictor of future relapse), we recently conducted a randomised, placebo-controlled trial in UC patients in clinical remission, but with high levels of faecal calprotectin (FC) (≥150 μg/g) at entry [96]. FC is a 36 kDa calcium- and zinc-binding protein, comprising up to 60% of the total cytosolic protein in granulocytes, which is stable in faeces for up to 7 days and correlates well with faecal granulocyte excretion. FC is a useful marker of mucosal inflammation in IBD patients and is able to predict clinical relapse 3–6 months before it occurs [97].

We aimed to define the effectiveness of 2 g/daily of EPA as FFA in achieving a 100-point reduction of FC at 6 months from baseline (primary endpoint) and in maintaining clinical remission (secondary endpoint) versus placebo (2 g/daily of capric and caprylic acids). Sixty patients were enrolled, and the primary endpoint was achieved in 19/30 (63.3%) versus 4/30 (13.3%) of patients in the EPA-FFA and placebo groups, respectively (OR 12, 95% CI: 3.12–46.24; *p* < 0.001). The secondary endpoint was achieved in 23/30 (76.7%) versus 15/30 (50%) of patients in the EPA-FFA and placebo groups, respectively (OR 3.29, 95% CI: 1.08–9.95; *p* = 0.035). No serious adverse events were documented. EPA-FFA decreased FC levels and appears to be a safe and promising treatment for maintaining symptom-free remission in UC patients.

#### Cochrane Reviews of n-3 PUFAs Treatment in IBD

Cochrane reviews have assessed the role of n-3 PUFAs in IBD, both in UC (Table 1) and in CD (Table 2).

Turner et al. [27] systematically reviewed the efficacy of n-3 PUFAs for maintaining remission in UC, with primary outcome being relapse rate during the observation time. Only 3 studies were included in the analysis for a total patient number of 138; these studies had different n-3 PUFAs formulations and doses, and none used “enteric-coated” formulations. No evidence was found in favour of n-3 PUFAs, but a need for further studies using “enteric-coated” capsules was highlighted.

De Ley et al. [28] systematically reviewed the efficacy of n-3 PUFAs for induction of remission in UC. The primary outcome was proportion of patients achieving remission within 3 months after treatment. Six studies were included in the analysis for a total of 159 patients. The authors concluded that existing information is insufficient to make recommendations on the use of fish oil for UC in clinical practice, and more research is required.

It is clear from these two Cochrane reviews in UC that the low number and poor quality of current studies in this field make it impossible to suggest guidelines on the use of n-3 PUFAs for treating patients with UC. As the authors stated in their conclusions, more large, well-designed studies, possibly including better active treatment and placebo preparations, are needed to address this important question.

Lev-Tzion et al. [13] systematically reviewed the efficacy of n-3 PUFAs for maintaining remission in CD. The primary outcome was relapse rate during the observation time, and six studies, for a total of 1039 patients, were included. The authors’ conclusions were based on the results of Feagan et al. [92]. Evidence from two large, high-quality studies suggests that n-3 PUFAs are probably ineffective for maintenance of remission in CD.

In our opinion, it is inappropriate to dismiss the potential of n-3 PUFAs to prevent relapse in CD at high risk of relapse on the basis of the EPIC studies; indeed, Turner et al. in a Cochrane systematic review [14] analysing the same six studies in CD (1039 patients) did not rule out a possible beneficial effect of n-3 PUFAs in these patients.

### 1.4. New Formulations and Bioavailability of n-3 PUFAs

The major natural dietary source of long-chain n-3 PUFAs is cold water-dwelling, oily fish; such fish can be consumed safely in large quantities.

The need to administer high doses of n-3 PUFAs to achieve a therapeutic benefit has encouraged the development of new formulations. One of the main concerns when increasing the daily dose of n-3 PUFAs is patient compliance. The commercially available marine fish-oil preparations are mainly in the form of fatty acid triglycerides or ethyl esters. It is clear that although fish oil has no serious toxicity, minor adverse events (AEs) such as dysgeusia, flatulence, pyrosis, halitosis, belching, and abdominal discomfort are common and may limit compliance [98]. Enteric coating of the fish-oil capsules may help to minimise upper gastrointestinal effects. There are conflicting data on the comparative bioavailability and AE profiles of n-3 PUFAs conjugated to a glycerol chain, as an ethyl ester conjugate and as FFA [99], but direct comparison of the three forms suggests that bioavailability is highest with the FFA conjugate and lowest with the ethyl ester conjugate [100].

Oral administration of fish oil containing the two main bioactive components, C20:5n3 EPA and C22:6n3 DHA, can replace C18:2n6 LA and C20:4n6 AA in a time- and dose-dependent manner in plasma and cellular phospholipid membranes [45]. Plasma n-3 PUFAs level is the easiest marker of EPA and DHA intake to use when measuring compliance with various fish-oil preparations. However, it is established that the plasma phospholipid fatty acid profile may change within a period of hours, depending on the type and timing of food intake [38]. Therefore, analysis of red blood cell (RBC) membrane n-3 PUFAs content is accepted as a more reliable measure [38]. The relatively long half-life of the RBC (120 days) provides a more stable measure of the incorporation of fatty acids into cellular phospholipid membranes [101]. The omega-3 index (the combined percentage content of EPA, DPA and DHA in RBC phospholipid membranes) can reach ≥8% with achievable and well tolerated n-3 PUFAs intake [38].

We recently carried out a pharmacokinetic study in a group of patients with IBD in stable remission and healthy volunteers, and showed that daily intake of 2 g EPA-FFA induces an efficient and consistent EPA incorporation into plasma phospholipids and RCB membranes. EPA-FFA is quickly converted into DHA via DPA, so that EPA can be considered as the “universal donor” of n-3 PUFAs. Treatment was very well tolerated, with only a few minor side effects [102].

There is no doubt that great interest exists in the development of a new formulation that can increase the bioavailability and long-term tolerability of n-3 PUFAs preparations.

TP-252 EPA-FFA is a new n-3 PUFAs enteric-coated powder preparation, in which two molecules of EPA in FFA form are bound to a scaffold made from mineral amino-acid. This preparation easily dissociates in aqueous media, displaying a favourable safety profile [103]. Maki et al. [104] reported on the superiority of MAT9001, a new n-3 PUFAs engineered-matrix, delayed-release 1 g capsule formulation to deliver EPA and DHA, versus ethyl-ester EPA in dyslipidaemia. A newly developed n-3 PUFAs hard tablet has shown faster absorption compared to traditional soft gelatine capsules, after testing for bioavailability of EPA and DHA [105]. Proposed reasons for the faster uptake of this preparation are the possibility of more rapid passage through the gastrointestinal system and improved solubility of fatty acids, reducing the dependence on emulsifying bile during absorption.

## 2. Conclusions and Future Perspectives

It is now clear that the Western diet often suffers from an important disequilibrium in the n-6/n-3 PUFAs ratio, reaching up to 20:1 in some cases, and that such an imbalance represents a powerful pro-inflammatory stimulus that can affect the onset of many underlying conditions, including IBD.

**Figure 3:**
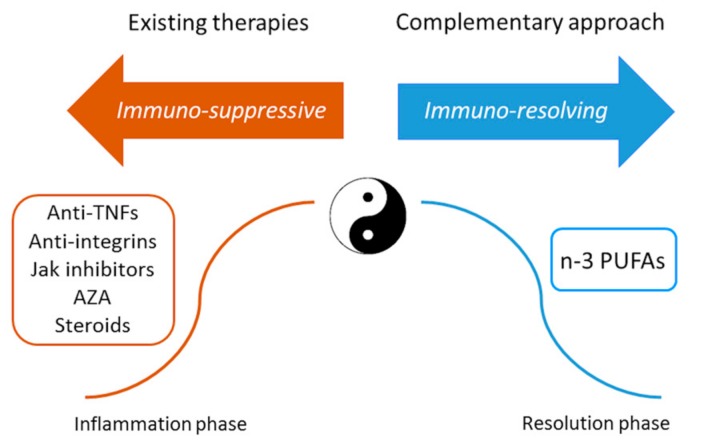
The immuno-suppressive and immuno-resolving approaches of inflammatory bowel disease treatment. AZA, azathioprine; PUFA, polyunsaturated fatty acid; TNF, tumour necrosis factor.

Since the 1980s, this disequilibrium has been corrected in patients with IBD by adding low-level n-3 PUFAs preparations to ongoing therapy. Subsequently, it was understood that positive results can be obtained by administering n-3 PUFAs pharmacologically, and hence increasingly sophisticated, high-concentration, and specific delivery technologies have been developed for this purpose. The discovery of n-3 PUFAs-derived anti-inflammatory molecules coined SPMs that can counteract and regulate pro-inflammatory chemical mediators, increase anti-inflammatory cytokines, and stimulate wound healing tissue regeneration and re-epithelialisation may offer a fascinating new complementary approach to IBD treatment. n-3 PUFAs therapeutic supplementation should be considered as an immune-resolving approach that can complement existing immunosuppressive therapies (Figure 3).

However, there remains a lack of well-designed clinical trials that include IBD patients selected using reliable criteria, such as new inflammatory disease markers (i.e., faecal calprotectin), to confirm the intriguing findings that are emerging from smaller studies, and, in particular, to confirm the possibility not only of preventing the recurrence of IBD but also of delaying or blocking its onset with the aid of a diet containing a balanced ratio of n-6/n-3 PUFAs.

## Figures and Tables

**Table 1:** Omega-3 fatty acids for maintenance and induction of remission in ulcerative colitis (UC).

Study	Study Type—Duration	Number of Subjects-Inclusion Criteria	Age (years)	Supplementation	Placebo	Concurrent Medications	Outcomes	Results *
**Hawthorne, 1992**	Double-blind, placebo-controlled, multicentre 1 year	34 adults: 19 (n-3 PUFAs arm) 15 (placebo arm) In remission or while recovering from relapse No diet restrictions	17–77	n-3 PUFAs as 10 mL liquid form twice daily of HiEPA (5 g/day EPA + 1.2 g/day DHA, as triglyceride concentrate)	Olive oil, 10 mL twice daily	5-ASA, steroids (≤20 mg/day)	Relapse rates (active symptoms and/or inflamed rectal mucosa)	42% vs. 48%; (*p* = 0.54)
							Time to relapse (days), median (IQR)	365 (265–365) vs. 349 (240–365) (*p* = NS)
**Loeschke, 1996**	Double-blind, placebo-controlled, multicenter2 years	64 adults: 33 (n-3 PUFAs arm) 31 (placebo arm) In remission or low disease activity (Gomez score <8)	n-3 arm: 39 ± 11 ^#^ Placebo arm: 40 ± 13 ^#^	Fish oil capsules, as long-chain ethyl esters n-3 PUFAs (5.1 g/day of total n-3 PUFAs; dose of EPA and DHA not reported)	Maize oil	5-ASA (discontinued 3 months after randomisation)	Relapse rate (≥4-point increase in Gomez score)	58% vs. 55%; (*p* = 0.81)
							Mean clinical disease activity score at the third month of treatment	2.2 vs. 4.4 (*p* < 0.05)
**Mantzaris, 1996**	Double-blind, placebo-controlled,single-centre 1 year	50 adults: 27 (n-3 PUFAs arm)23 (placebo arm) Clinical, endoscopic remissionNo diet restrictions	17–65	n-3 PUFAs as 10 mL liquid form twice daily of MaxEPA (3.2 g/day EPA + 2.1 g/day DHA, as triacylglycerol)	Olive oil, 10 mL twice daily5-ASA	5-ASA (3.6 g/day)	Relapse rate (active symptoms or endoscopic signs of relapse)	27% vs. 28% (*p* = 1.0)
							Time to relapse (days), median (IQR)	235 (100–365) vs. 218 (79–365) (*p* > 0.1)
**Aslan, 1992**	Double-blind, placebo-controlled;crossover design 8 months (3 intervention period + 2 washout period)	17 adultsMild to moderate disease activity	31–74	MaxEPA capsules (2.7 g/day EPA + 1.8 g/day DHA)	Corn oil (10.3 g OA + 2.1 g PA + 1.8 g LA)	Oral steroids (<20 mg/day) or SASP	Clinical response (decrease in DAI score after MaxEPA)	56% vs. 4% *p* < 0.05
							Steroid-sparing effects during MaxEPA therapy	72% reduced anti-inflammatory dosage and/or eliminated steroids (*p* value not reported)
**Stenson, 1992**	Double-blind, placebo-controlled, randomised, multicentre; crossover design 5 months (4 intervention period + 1 washout period)	24 adults Active disease	25–62	MaxEPA capsules (3.24 g/day EPA + 2.16 g/day DHA, as triacylglycerol)	Vegetable oil (12.36 g OA + 2.52 g PA + 2.16 g LA)	Prednisone, SASP	Endoscopic score improvement	n-3 PUFAs group: mean decrease of −2.09 (95% CI: −4.63 to 0.45; *p* = 0.06) Placebo group: mean decrease of −0.17 (95% CI: −1.75 to 1.41; *p* = 0.10)
							Steroid-sparing effect	NS in both groups
**Stack, 1997**	Double-blind, controlled, randomised; parallel design 4 months	66 adults: 14 (n-3 PUFAs group) 13 (n-6 PUFAs group) 13 (n-3 and n-6 PUFAs group) 13 (placebo group)		n-3 PUFAs group: 1.5 g EPA	n-6 PUFAs group: 2.1 g GLA Placebo group: sunflower oil	Steroids	Steroid-sparing effect	No evidence with n-3 or n-6 PUFAs compared to placebo either alone or in combination (*p* value not reported)
**Almallah, 1998**	Double-blind, placebo-controlled, randomised, single-centre; parallel design 6 months	18 adults: 9 (n-3 PUFAs arm) 9 (placebo arm) Low disease activity (Gomez score < 8) On Western diet	n-3 PUFAs arm: 29–64Placebo arm: 32–72	n-3 PUFAs as 15 mL fish-oil extract (3.2 g/day EPA + 2.4 g/day DHA)	Sunflower oil, 15 ml (2.6 g/day oleic acid + 7.9 g/day linoleic acid)	SASP or 5-ASA	Clinical response	*p* < 0.05
							Endoscopic score improvement (RSS)	*p* = 0.013 (n-3 PUFAs arm) *p* = NS (placebo arm)
							Histological score improvement	*p* = 0.016
**Dichi, 2000**	Randomised, single-centre; crossover design 6 months (2 intervention period + 2 washout period + 2 crossover intervention period)	10 adults Mild to moderate disease activity	33–65	5.4 g/day n-3 PUFAs fish-oil capsule, as fatty acids (180 mg EPA + 120 mg DHA in each capsule)	2.2 g/day SASP		Laboratory blood parameters	↑ of CRP, ERS, PLT during n-3 PUFAs treatment (*p* < 0.01) Changes during SASP therapy: *p* = NS
							Improvement in sigmoidoscopy score	At entry, mean 9.6 (SD ± 2.8) After n-3 PUFAs treatment, mean 5.0 (SD ± 5.7) *p* < 0.01
**Varghese, 2000**	Double-blind, randomised; parallel design 6 months	51 adults: 21 (n-3 PUFAs arm) 30 (placebo arm) Active and extensive disease		Fish oil 5.6 mg/day	Sunflower oil		Clinical scores improvement	n-3 PUFAs arm: *p* = 0.001 (no quantitative data presented)
							Endoscopic score improvement	n-3 PUFAs arm: *p* = 0.054 (no quantitative data presented)

* Expressed as results in the n-3 PUFAs arm versus results in the placebo arm in studies investigating omega-3 fatty acids for maintenance of remission in UC. ^#^ Age expressed as mean ± SD. 5-ASA, 5-aminosalicylic acid or mesalazine; CI, confidence interval; CRP, C reactive protein; DAI, disease activity index; DHA, docosahexanoic acid; EPA, eicosapentanoic acid; ERS, erythrocyte sedimentation rate; GLA, gamma linolenic acid; IQR, interquartile range; LA, linoleic acid; NS, not significant; OA, oleic acid; PA, palmitic acid; PLT, platelet; PUFA, polyunsaturated fatty acid; RSS, rectosigmoidoscopy; SASP, sulfasalazine; SD, standard deviation; UC, ulcerative colitis.

**Table 2:** Omega-3 fatty acids for maintenance of remission in Crohn’s disease (CD).

Author	Study Type-Duration	Number of Subjects-Inclusion Criteria	Age (years)	Supplementation	Placebo	Concurrent Medications	Outcomes	Results (n-3 PUFAs Arm vs. Placebo arm)
**Belluzzi, 1996**	Double-blind, placebo-controlled, single-centre 1 year	78 adults: 39 (n-3 PUFAs arm) 39 (placebo arm) CDAI < 150 at baseline, but at high risk of relapse No diet restrictions	18–67	Enteric-coated, time-released, fish-oil capsule (1.8 g/day EPA + 0.9 g/day DHA, as FFAs)	Capsules 500 mg Miglyol 182 (caprylic acid + capric acid)	None	Relapse rate (CDAI >150 or ≥100 points increase in CDAI from baseline)	11.28% vs. 27.69% (*p* < 0.001)
							Time to first relapse	>1 year vs. 4 months (log rank test 0.006)
							Adverse events	10.3% vs. 2.6% (diarrhoea)
**Lorenz-Meyer, 1996**	Double-blind, placebo-controlled, multicenter 1 year	135 adults: 70 (n-3 PUFAs arm) 65 (placebo arm) CDAI < 150 at baseline (steroid-induced remission after an acute relapse) Diet low in AA and rich in fibre	17–65	Non-enteric-coated n-3 PUFAs capsules (3.3 g/day EPA + 1.8 g/day DHA, as ethyl ester)	Corn oil	Low-dose prednisolone in the first 8 weeks	Relapse rate (CDAI >200 or ≥60 points increase in CDAI from baseline, plus ≥2 SD above normal mean CRP increase	57.00% vs. 36.55% (*p* = 0.84)
							Time to first relapse	159 days vs. 133 days (NS)
							Adverse events	1.4% vs. 1.5% (diarrhoea) 14.3% vs. 3.1% (halitosis) 14.3% vs. 3.1% (upper GI symptoms)
**Belluzzi, 1997**	Double-blind, placebo-controlled, single-centre 1 year	50 adults; 26 (n-3 PUFAs arm) 24 (placebo arm) CDAI <150 one month after ileal resection		Enteric-coated, time-released, fish-oil capsule (1.8 g/day EPA + 0.9 g/day DHA, as FFAs)	Capsules 500 mg Miglyol 182 (caprylic acid + capric acid)	None	Clinical relapse rate (CDAI >150 with an increase of >50 points from baseline) Histological relapse	2.8% vs. 5.21% (*p* = 0.24) 9.34% vs. 15.62% (*p* = 0.09)
							Adverse events	None (personal communication)
**Romano, 2005**	Double-blind, placebo-controlled, multicenter 1 year	38 children: 18 (n-3 PUFAs arm) 20 (placebo arm) PCDAI <20 for at least 2 months at baseline	5–16	Enteric-coated n-3 PUFAs capsules (1.2 g/day EPA + 0.6 g/day DHA, as triglycerides)	Olive oil	Time-dependent 5-ASA (50 mg/kg/day)	Relapse rate (PCDAI > 20)	61% vs. 95% (*p* < 0.001)
							Time to first relapse	8 months vs. 1 month
							Adverse events	None
**Feagan, 2008 (EPIC-1)**	Double-blind, placebo-controlled, multicenter 52 weeks	363 adults: 183 (n-3 PUFAs arm) 180 (placebo arm) CDAI <150 for at least 3 months but <12 months at baseline	n-3 PUFAs arm: 40.5 ±15.2 ^#^ Placebo arm: 38.2 ± 13.1 ^#^	Enteric-coated, time-released n-3 PUFAs capsules (2.2 g/day EPA + 0.8 g/day DHA, as FFAs) *	4 g/day medium-chain triglyceride oil	None	Relapse rate (CDAI >150 or >70 points increase from baseline)	31.6% vs. 35.7% HR 0.82, 95% CI: 0.51–1.19 (*p* = 0.30)
							Adverse events	18.7% vs. 11.4% (diarrhoea)1% vs. 0.5% (dysgeusia) 9.1% vs. 2.2% (nausea) 14.4% vs. 6.5% (all upper GI symptoms)
							Change in mean CDAI	NS
							Change in SF-36 scores	NS
**Feagan, 2008 (EPIC-2)**	Double-blind, placebo-controlled, Multicenter 58 weeks	375 adults: 187 (n-3 PUFAs arm) 188 (placebo arm) CDAI < 150 after 8 weeks of steroid-induced remission	n-3 PUFAs arm: 38.5 ±13.8 ^#^ Placebo arm: 40 ± 13.6 ^#^	Enteric-coated, time-released n-3 PUFAs capsules (2.2 g/day EPA + 0.8 g/day DHA as FFAs) *	Four 1 g of medium-chain triglyceride oil	Prednisone 20 mg/day or budesonide 6 mg/day tapered off over 8 weeks from randomisation	Relapse rate (CDAI >150 or >70 points increase from baseline)	47.8% vs. 48.8% HR 0.90, 95% CI: 0.67–1.21 (*p* = 0.48)
							Adverse events	23.2% vs. 19.7% (diarrhoea) 5.3% vs. 1.1% (dysgeusia) 15.9% vs. 10.1% (nausea) 35.4% vs. 23.9% (all upper GI symptoms)
							Change in mean CDAI	NS
							Change in SF-36 scores	NS

* Dose of the study drug increased by 1 g every week to improve tolerability, till the final dose (2 capsules twice daily). ^#^ Age expressed as mean ± SD. 5-ASA, 5-aminosalicylic acid or mesalazine; AA, arachidonic acid; CDAI, Crohn’s disease activity index; CI, confidence interval; CRP, C reactive protein; DHA, docosahexanoic acid; EPA, eicosapentanoic acid; FFA, free fatty acid; GI, gastrointestinal; HR, hazard ratio; PCDAI, Pediatric Crohn’s disease activity index; PUFA, polyunsatured fatty acid; NS, not significant; SD, standard deviation; SF-36, 36-Item Short-Form Health Survey.

## Abbreviations

5-ASA	5-aminosalicylic acid
AA	Arachidonic acid
AE	Adverse event
ALA	α-linolenic acid
CD	Crohn’s disease
CI	Confidence interval
COX	Cyclooxygenase
CDAI	Crohn’s Disease Activity Index
CRP	C reactive protein
DHA	Docosahexaenoic acid
DPA	Docosapentaenoic acid
EET	Epoxyeicosatrienoic acid
ELOVL	Elongase of very long chain fatty acids
EPA	Eicosapentaenoic acid
EPIC	European Prospective Investigation into Cancer and Nutrition
FADS	Fatty acid desaturase
FC	Faecal calprotectin
FFA	Free fatty acid
GLA	n-6 gamma linolenic acid
HETEs	Hydroxyeicosatetraenoic acid
HR	Hazard ratio
IBD	Inflammatory bowel disease
IL	Interleukin
LA	Linoleic acid
LOX	Lipoxygenase
LT	Leukotriene
LX	Lipoxin
MAPK	Mitogen-activated protein kinase
NFkβ	Nuclear factor kB
NLRs	NOD-like receptors
NLRP3	NOD-like receptor protein 3
NO	Nitric oxide
NOD2	Nucleotide-binding oligomerisation domain 2
NOS	Nitric oxide synthase
OA	Oleic acid
OR	Odds ratio
PG	Prostaglandin
PPAR-γ	Peroxisome proliferator-activated receptor γ
PUFAs	Polyunsaturated fatty acids
RBC	Red blood cell
SNPs	Single nucleotide polymorphisms
SPM	Specialised pro-resolving mediator
TLRs	Toll-like receptors
TNFα	Tumor necrosis factor α
TX	Thromboxane
UC	Ulcerative colitis
VCAM-1	Vascular cell adhesion molecule 1
